# Documenting HIV research-utilization activities, outputs and outcomes: examples and lessons learned from Project SOAR

**DOI:** 10.1186/s13690-021-00628-x

**Published:** 2021-06-15

**Authors:** Samuel Kalibala, Irit Sinai, Tara Nutley

**Affiliations:** 1 Project SOAR, 4301 Connecticut Ave., NW, Suite 280, Palladium, Washington, DC, USA; 2 Data Informatics and Analytical Solutions, Palladium, Washington, DC, USA; 3 Data Informatics and Analytical Solutions, Palladium, Chapel Hill, North Carolina USA

**Keywords:** Research utilization, Logic model, Implementation science, HIV/AIDS, Measuring research outcomes

## Abstract

The importance of using research findings to inform policy and program decisions is well recognized, but the literature on measuring research utilization activities is scarce. As funding to support some areas of research wanes or remains stagnant, the need to document the value of investing in research by its’ effect on improved programs and policies becomes increasingly necessary. We present the experience of Project SOAR, a six-year USAID-funded project focusing on HIV/AIDS-related implementation research, to demonstrate measurement of research utilization. We follow the project’s research-utilization logic model, including inputs, activities, outputs, and outcomes. We present tools the project developed and examples from project studies and discuss what works, remaining challenges and how to overcome them, and lessons learned. We then make recommendations for incorporating research-utilization activities and measurement in implementation-research studies.

## Background

Research utilization is “the implementation of research-based knowledge (science) in practice” [1]. The United States Agency for International Development (USAID) supports implementation research to identify, develop and measure the impact of innovative strategies to improve public-health service delivery, and to inform policies and programs [2]. However, the link between research findings and public-health programs is often weak. While public-health services and programs usually claim to be evidence based, frequently relevant research findings are not considered in their design and implementation, and some studies are undertaken without considering programmatic implications [3]. Research utilization is the process of synthesizing research findings and using them to inform policy or program decision making.

The importance of research utilization is well recognized, and there is substantial literature documenting the implementation of research-utilization activities. However, not much is known about measuring the outcomes of such activities [4]. Validated tools for measuring research use are scarce. In a literature review of measuring research utilization, Estabrooks and Wallin [1] noted that researchers who have measured research use have tended to develop their own tools. Straus et al. [3] cite a comprehensive literature review and conclude that the most common methods for measuring research utilization rely on self-reporting by investigators or stakeholders.

This manuscript adds to the limited but growing body of knowledge on how to measure outcomes of research utilization, by presenting research-utilization activities undertaken by Project SOAR (Supporting Operational AIDS Research) a six-year implementation-science project (2014–2020), funded by USAID. The project was designed to improve HIV service delivery by conducting high quality research to meet data needs of stakeholders, strengthen the capacity of stakeholders to conduct implementation-science research and use study findings to guide planning, funding and program implementation. The project consisted of more than 50 research studies in 24 countries, focusing on implementation science to improve HIV prevention, care and treatment services. Research utilization was embedded in all of these studies and was incorporated in all phases of study implementation, starting with the study design. That is, studies were designed with the end in mind – they were designed to yield data that will be of use to policy decision makers.

We document how the project’s research-utilization activities were measured, and lessons learned in documenting inputs, outputs, and outcomes of research-utilization activities, including key challenges and recommendations for overcoming them. We do so by following the project logic model.

## Measuring Project SOAR use of research findings

### Project SOAR research-utilization logic model

Because the focus of Project SOAR was implementation science and the utilization of project research findings, the project developed a research-utilization logic model – a road map for those project activities that specifically focus on research utilization. In developing the research-utilization activity logic model, Project SOAR utilized The Centers for Disease Control and Prevention (CDC) guidelines for logic models of evaluation programs designed to address sexually transmitted infections (STI). The guidelines outlined the following components of a logic model [5]:

- Inputs (resources): funding, staff, materials;
- Activities (program, events or strategies): staff training, patient testing and treatment;
- Outputs (products of activities): number of patients treated, quality of training;
- Short-term outcomes (intended effects that occur within weeks or months): changes in knowledge, skills, or beliefs; increased proportion of patients treated;
- Intermediate outcomes (intended effects that occur over the mid-term: months-years): changes in policies or behaviors, increased proportion of sexual partners treated, increased condom use; and
- Long term outcomes or impact (long term intended effects: years or decades): reduced STI prevalence; changes in morbidity or mortality

**Fig. 1:**
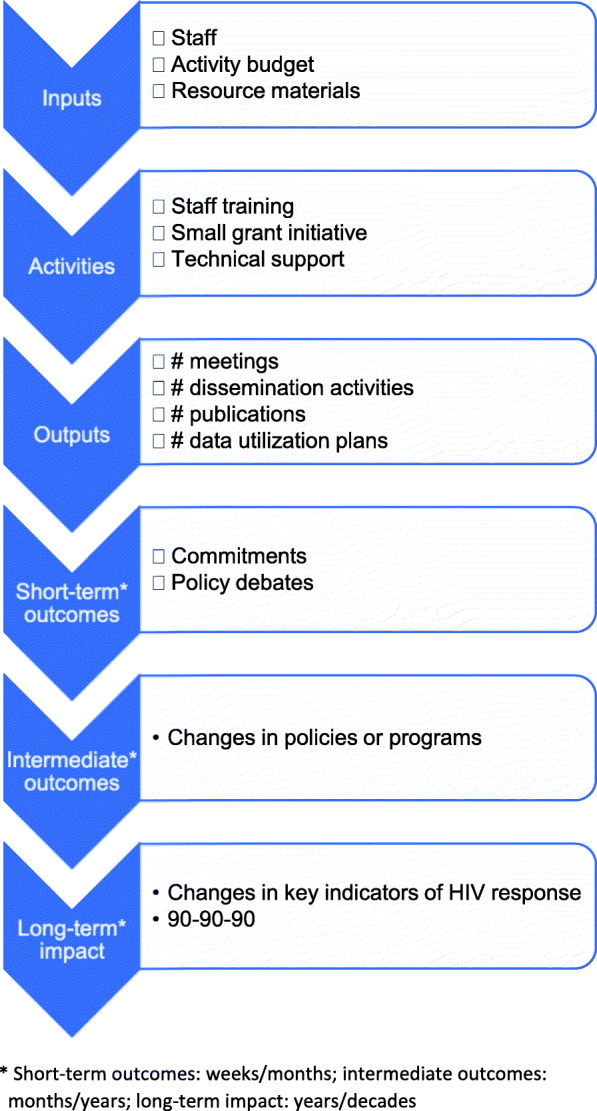
Logic model for Project SOAR research utilization activities

While CDC’s logic model guidelines were intended for use in monitoring and evaluating STI programs (not research), these components are widely accepted as key elements of most logic models. Therefore, Project SOAR adopted the CDC’s logic model, but modified it to fit a different purpose. Project SOAR’s inputs into research-utilization activities are comprised of staff time, funding for travel, meetings and workshops; and guidance documents and tools to facilitate activity implementation. Outputs of these activities are the number of data use plans, meetings and dissemination events conducted by study teams to engage stakeholders in study implementation and dissemination of results. Outcomes are defined as the use of study findings, by stakeholders, to improve services, service guidelines and policies, with the ultimate long-term impact being improvements in the Joint United Nations Programme for HIV/AIDS (UNAIDS) 90–90-90 goals, which call for 90% of HIV-infected individuals to be diagnosed by 2020, 90% of whom will be on anti-retroviral therapy (ART) and 90% of whom will achieve sustained virologic suppression. The Project SOAR research-utilization logic model is shown in Fig. 1.

### Measuring research-utilization inputs

Research-utilization inputs include staff, budget, and resource materials. Project SOAR employed three staff persons dedicated to research utilization:

1. A full time Research-Utilization Advisor, who served as a dedicated knowledge broker;
2. A full time Science Writer; and
3. A Knowledge Management Specialist, who worked on research utilization 50% of time.

Their roles are described in the section on Activities below. With guidance from these dedicated staff, all Project SOAR’s principal investigators and staff considered research utilization in all aspects of their research studies. For each study, the principal investigator included a budget allotment for research utilization activities. In addition, at the global level, the budget included allotments for capacity strengthening workshops, small grants for research utilization, as well as travel for the Research-utilization advisor, Science Writer and Knowledge Management Specialist.

The project developed and used a Research-Utilization Process Guide, which provided guidelines and tools to use by project partners, researchers, and other staff [6].

### Measuring research-utilization activities

By definition, research utilization involves policy makers and other stakeholders, such as government officials, program managers, academia and community leaders, who may use research findings to inform policies and programs, and who may have need for such data. While engaging key stakeholders in study design, and sharing knowledge of study findings with them, may not be effective on their own to change practices or policies, they are necessary pre-requisites for change [4]. Therefore, Project SOAR conducted activities to strengthen the capacity of stakeholders to generate, analyze and use research findings. During site-selection exploratory visits to study countries, each study’s principal investigator identified in-country colleagues to act as co-principal investigators on the studies, as well as key stakeholders to help refine research questions to address priority policy and program needs in the country, and undertake capacity-building activities, to promote their demand for and use of research data. One such activity was convening two regional capacity-strengthening workshops in Johannesburg, South Africa in February, 2017 and May, 2018. The workshops were attended by 48 key stakeholders from 12 countries in which the project was active, and focused on skill building around demand for research data and use of findings.

To enable workshop participants to apply the skills they learned to generate and use data, Project SOAR posted a request for proposals to in-country researchers and issued small grants of not more than $10,000 each to nine of the 16 applications received. Using these funds, in-country researchers conducted secondary analyses of available data to address locally relevant research questions, convened stakeholder meetings to disseminate findings, and submitted conference abstracts and journal manuscripts.

While studies funded through the small grants’ mechanism yielded interesting and utilized findings, the main focus of Project SOAR was the studies undertaken directly by the project. The project recognized the importance of engaging stakeholders in all stages of each of these studies, from design, through implementation, to ensure that the research question has policy and program relevance, and to maximize the eventuality that findings are used to inform activities. Therefore, the engagement with stakeholders started in the inception phase of each study, during which the study design and methodology were discussed, and continued into later phases of the study. During study implementation researchers shared preliminary, and eventually final, study findings with stakeholders, and brainstormed with them implications of the findings to current policies and programs.

To document stakeholder engagement in each study, Project SOAR developed a Stakeholder Engagement Template. An example of how the template was used is shown in Table 1, in which the template is populated with information from the Lesotho IMPROVE study [7]. The template was completed by the principal investigator of each Project SOAR study and submitted to project management twice per year, as part of the project management and monitoring. The template describes all stakeholder engagement efforts for the study, including which stakeholders were engaged, dates and modes of engagement, as well as what was discussed during the engagement.

**Table 1:** Stakeholder engagement template, Lesotho IMPROVE study [7]

Stakeholder(s)	Date of engagement	Mode of engagement	What was discussed	Research utilization outcomes of the meeting
Wide range of stakeholders including District Health Management Teams, leaders of people living with HIV, leaders of village health workers, Global Fund, PEPFAR, UN agencies and implementing partners.	February 2015	Face-to-face inception meeting convened by the Lesotho Ministry of Health technical working group for Prevention of Mother to Child Transmission	EGPAF Lesotho team presented study concept and outline	• Meeting participants gave input in site selection • Three Ministry of Health researchers joined the study team as investigators, including one as co-Principal Investigator • Ministry of Health technical working group for Prevention of Mother to Child Transmission reviewed the final study proposal 2 months later.
District Health Management Team meeting, Scott District	March 2017	Face-to-face meeting at District offices	Baseline study findings showing insufficient number of village health workers	District Health Management Team selected, trained and deployed 46 new village health workers
Lesotho Ministry of Health technical working group for Prevention of Mother to Child Transmission	October 2018	Face-to-face meeting at the Lesotho Ministry of Health	Study progress, including key early lessons	• The head of the Family Health division at the Ministry of Health expressed a great interest in the different aspects of community-based support; • She communicated her plan to use the lessons learned to revive the National Village Health Workers program; and • Members of the technical working group expressed interest in adopting key aspects of the intervention to include in routine maternal and neonatal child Health services at the end of the study.
Facility leadership, including staff	November and December 2018	Face-to-face onsite meetings at each of the 12 facilities that participated in the study	Facility-specific update on study implementation progress	Each participating health facility decided on strategies to address gaps identified by the study in addressing retention and challenges in follow up.

Researchers also engaged stakeholders through existing technical working groups or study-specific research advisory committees, using presentations and results briefs to present study findings. Each Project SOAR study conducted one research advisory committee meeting at inception, another to share preliminary study results, followed by a data interpretation meeting to discuss final study findings and determine study recommendations, before conducting a final study dissemination meeting, attended by a broader group of stakeholders. In each case the researchers reported to Project SOAR management the key aspects of the meeting using the template in Table 1. The data in the last column of the table, or outcomes of each stakeholder engagement, then flowed directly into outcome monitoring, described later. Further details of the process of engagement of stakeholders in Project SOAR studies are provided in a previous publication [8].

The Research-Utilization Advisor also provided technical support to study teams by way of country visits, phone calls and e-mails, and supported the formation of country-level research advisory committees in each of the countries where Project SOAR was active. The technical support included review of study proposals, meeting plans, agendas, and power point presentations; as well as assisting in selecting the types of stakeholders to be engaged. By the end of the fifth project year, Project SOAR had established 54 research advisory committees and supported them in developing 46 data-use plans in 24 countries. Members of Project SOAR’s scientific team supported in-country teams by reviewing their draft publications and by mentoring small grant recipients. The Research-utilization advisor, the Science Writer and the Knowledge Management Specialist also supported the researchers in editing, formatting and preparing publications, ensuring that the publications highlighted programmatic and policy implications of study findings.

Other activities undertaken by the Research-Utilization Advisor included promoting research utilization among project researchers and staff and providing technical assistance to monitoring how project findings had been utilized for program and policy change. He provided technical input to study protocols to ensure that study outcomes were aligned with salient issues in programs and policies of the country where the research was being conducted, and collaborated with principal investigators in study protocol-development and trips to study countries, where they scoped the landscape of stakeholders and policy issues relevant to the research topic of interest.

The Research-Utilization Advisor worked with researchers to strengthen the capacity of in-country investigators and governmental stakeholders to access, review and use research to improve programs and policies through workshops and small grants. In addition, the advisor and Project SOAR’s Science Writer and Knowledge Management Specialist worked with researchers to analyze the data, identify key findings, develop practical recommendations and create power point presentations, activity briefs and results briefs that researchers used to disseminate their research and results. Table 2 summarizes research-utilization activities of Project SOAR through the end of the fifth project year.

**Table 2:** Project SOAR activities to facilitate research utilization

Activity Type	Achieved by end of Yr-5	Details
Research utilization capacity-strengthening workshops for in-country researchers and stakeholders	2 four-day regional workshops in Johannesburg, South Africa	Feb 2017: 28 participants, from 12 countries May 2018: 20 participants from 8 countries.
Funding of small grant proposal submitted by in-country researchers	16 applications received, 9 selected and funded	About $10,000 per grant
Technical support visits by the Research-Utilization Advisor	45 visits to 9 countries	
Formation of Research Advisory Committees	54 (Out of 58 studies initiated)	A few nested studies shared the Research Advisory Committee of the parent study

### Measuring research-utilization outputs

As shown above, Project SOAR conducted technical support and capacity strengthening activities directed at in-country research teams and stakeholders, to enable them to carry out research-utilization activities. Outputs of these activities were progressively monitored for each study as it was ongoing, then tabulated project wide. The outputs for Project SOAR through the end of the fifth project year are shown in Table 3. These outputs were directly linked to Project SOAR’s inputs and activities.

**Table 3:** Outputs: Project SOAR research utilization outputs

Activity Type	Achieved by end of the fifth project year	Remarks
Study-specific data-use plans to guide dissemination of results locally	46 (Out of 58 studies)	Global studies did not submit in-country data-use plans
Activity Briefs	58	One for each study
Results Briefs	74	Some studies produced more than one results brief
Joint national research-advisory group meetings in countries with multiple SOAR studies	11 One-day in-country meetings of about 40 participants each	• Three in Malawi: July 2017, September 2018, November 2019 • Three in South Africa: May 2017, September 2018, November 2019 • Two in Tanzania: March 2017, November 2019 • One in Uganda: February 2019 • One in Kenya: February 2019 • One in Zambia: February 2019
Briefings of national directors of AIDS programs and AIDS commissions	6 boardroom meetings at national AIDS program/council offices lasting about 2 h each.	• Uganda: Aug 2018 • South Africa: Nov 2019 • Malawi: Oct 2018 • Kenya: Feb 2019 • Tanzania: May 2018 • Zambia: Feb 2017
Meetings (including webinars and informal briefings) convened with USAID and/or other stakeholders to share interim results from SOAR studies	116	
Oral/poster presentations by SOAR principal investigators at international, regional, and national conferences	111	
Manuscripts submitted to peer reviewed journals	59	
Presentations sharing Best Practices from SOAR’s research utilization approach	Four Technical Expert meetings	• Makerere University Medical School, Uganda: August 2018, 60 faculty members and researchers • Washington, DC Technical Advisory Network: May 2019, 50 research utilization experts • Mexico City, International AIDS Society (IAS) Conference: July 2019, Satellite meeting on research utilization • Kigali, Rwanda, International Conference on AIDS and STIs in Africa (ICASA): December 2019 Satellite meeting on research utilization
Publications sharing best practices from Project SOAR research-utilization approach	Four	• Blog on Capacity Strengthening on USAID website • Q&A on Project SOAR website • Two Journal articles in AIDS and Behavior

The research advisory committees developed data-use plans that they used to engage stakeholders throughout each study as living documents, modified as they gained more knowledge about the landscape of stakeholders and priority program and policy issues. Final data-use plans, developed at the final dissemination meeting of each study, were plans for continued engagement of stakeholders after the close of Project SOAR. As part of the ongoing data-use plan, research advisory committees identified and coached program and policy influencers, referred to as champions, to continue engaging stakeholders in various forums to integrate study findings into decision making processes.

As shown in Table 3, Project SOAR researchers developed 58 Activity Briefs and 74 Results Briefs. Briefs are two-page documents used to disseminate study information to stakeholders. Activity briefs focus on one study, stating the study objectives, methods, and proposed research-utilization process; Result briefs focus on key findings, programmatic implications and recommendations. The briefs were printed and shared with stakeholders as hard copies and were also published on the Project SOAR website.

In the six countries where the project had multiple studies, Project SOAR researchers conducted 11 joint research advisory group meetings that brought together an average of 40 study staff and key stakeholders in each country, to interpret preliminary and final study findings, identify key messages, and make practical and actionable recommendations that applied study findings to strengthen policies and programs. In countries where it was not possible for national authorities to attend dissemination meetings, Project SOAR researchers requested boardroom meetings at national AIDS program offices, where they shared study findings with top-level government officials and discussed policy and programmatic implications of the findings.

Beyond national meetings, Project SOAR researchers shared interim research findings in 116 webinars and meetings convened by USAID and other stakeholders, and in 111 oral and poster presentations in regional and international AIDS conferences. They also submitted 59 manuscripts for publication in peer reviewed journals, to further disseminate study findings. As Table 3 shows, the project compiled and shared best practices of the project’s research-utilization approach to communities of practice through meetings and publications [8–11].

### Measuring research-utilization outcomes

Outcomes of stakeholder engagement are incidents where study findings are used to improve policies and programs. The literature suggests at least three domains of research use, where ‘use’ refers to an event or action by stakeholders to change programs or policies based on study findings [4, 12, 13]. *Instrumental Use* is when stakeholders use research to make policy or program decisions. *Conceptual Use* is when stakeholders apply the new knowledge in their thinking and conceptual understanding of the issue [13] and use study findings in debates and ‘public and professional discourses’ [12] without necessarily taking action to change policy or practice as a result of the study findings. Finally, *Persuasive Use* is when stakeholders use research data to influence or persuade other stakeholders, such as politicians to pass a bill or community members to change a behavior.

When Project SOAR researchers shared research information, updates on the research process, or study findings, some stakeholders made comments and asked questions to seek clarification, interpret the data and draw programmatic or policy implications. These productive interactions, or instances of knowledge exchange, are often viewed as crucial *Conceptual Use* that leads to *Instrumental Use* [14] and should be documented [4]. Indeed, Penfield et al. [15] recommend documentation of the bi-directional flow of knowledge between researchers and stakeholders. Project SOAR considered conceptual and persuasive use of research findings as short-term outcomes (weeks/months), and instrumental use as intermediate outcomes (months/years).

#### Project SOAR short-term outcomes

In the bi-directional exchange between researchers and stakeholders some stakeholders may commit to implement study recommendations. These commitments can be classified as *Conceptual Use*, even if they are only verbal, and it was important for Project SOAR study teams to record them as prompts for future follow up. Indeed, Project SOAR champions, identified in the data-use plans (Table 3) planned to follow up to verify whether the stakeholders fulfilled these commitments; and if there were barriers to fulfilling the commitments, how they could be addressed.

Several stakeholders stated a commitment to use the study intervention in some way, such as integration into routine service delivery, pilot-testing an intervention, or taking an intervention to scale based on research findings. Other stakeholders committed to addressing service gaps identified by the study, for example through improved program monitoring and evaluation or through training. These are summarized in Table 4 [16–22]. It is important to note that in some situations, while no policy or program change were affected at the time of reporting, Project SOAR recorded these commitments and considered them to be a step in the right direction. For example, in the Zambia project YES study, the integration of anti-stigma activities in the study intervention was a step towards developing an anti-stigma program [17]. Similarly, in the Kenya and Uganda Pediatric Case Finding studies, the development of quality improvement plans was a step towards improved programing [16].

**Table 4:** Project SOAR Short-term outcomes (weeks- months)

Name of study	Key Finding	RU Outcome
**Commitments and policy debates**		
Kenya and Uganda Pediatric Case Finding [16]	Missed opportunities for prevention of mother to child transmission of HIV (PMTCT)	Program implementers developed quality improvement plans to address gaps
Zambia Project YES [17]	High levels of stigma for people living with HIV Transitioning youth on ART to adult care is feasible	Investigators integrated anti-stigma components in the study intervention A separate non-governmental organization expressed desire to adapt and use the study’s intervention in their program
Uganda PEPFAR Geographical Pivot [18]	Withdrawing PEPFAR support from some health facilities in Uganda was not followed immediately by Uganda government support as expected	Policymakers reacted by debating the country’s preparedness for the possible reduction in donor funding for ART
Tanzania gender-based violence [19]	A gender-based violence intervention in study facilities is feasible to implement	The Tanzania Ministry of Health expressed interest in integrating the intervention into routine health care
Namibia TnS [20]	While the test and start program was feasible to implement, there was lack of patient understanding of viral loads	The Namibia Ministry of Health committed to developing and pilot-testing a viral-load literacy intervention for patients on ART
Tanzania FSW/FP [21]	A proportion of women on ART expressed a desire for safer conception	The Tanzania Ministry of Health and implementing partners committed to strengthening the skills of staff in counseling on safer conception
Uganda DISCO [22]	The disclosure intervention tested in the study was highly efficacious among youths	Policymakers expressed interest in rolling out the intervention nationally
**Policy Influence**		
Global Fund TA [23]	It is feasible and useful to analyze and deposit HIV prevalence and incidence data for key population in a data repository	Global Fund, UNAIDS and CDC retrieved data from the repository for use in policy decision making
Malawi DREAMS [24]	High prevalence of herpes simplex among adolescent girls and young women	The Malawi Ministry of Health epidemiologist confirmed that the data were vital for informing the ongoing go/no-go national discussions about the use of PrEP among adolescent girls and young women

Other than commitments, another *Conceptual Use* of research can be an improvement in knowledge about a topic that feeds into policy debate. For example, the Uganda PEPFAR (President’s Emergency Plan For AIDS Relief) Geographical Pivot study showed that when PEPFAR transitioned out of some health facilities, the government was not immediately able to provide continuation of ART [18]. This finding elicited a debate among senior policy makers about the government’s preparedness for donor withdrawal.

Table 4 also shows examples of *Persuasive Use* [23, 24]. In the case of the Malawi DREAMS (Determined, Resilient, Empowered, AIDS-free, Mentored, and Safe) study, the Ministry of Health Department of HIV/AIDS epidemiologist intended to use study findings to influence the ongoing go/no-go discussions about Pre-Exposure Prophylaxis (PrEP) use in this population; and the organizations retrieving data from the Global Fund repository on key populations, intended to use the data to influence global policy and interventions [24].

#### Project SOAR intermediate outcomes

Some stakeholders made policy or program decisions and acted on these decisions based on Project Soar study findings. This is *Instrumental Use*. Table 4 shows five instances of instrumental use of Project SOAR study findings. For example, in Tanzania after learning that community-based provision of ART improved initiation and retention in treatment among key populations, the Ministry of Health authorized community-based ART services for these populations. All service delivery providers were informed of this change in a formal government circular. Similarly, in Senegal, the AIDS program added HIV self-testing to the national strategy based on findings from a Project SOAR feasibility study (Table 5) [25–29].

**Table 5:** Project SOAR Short-term outcomes (weeks- months

Name of study	Key Finding	Research utilization outcome
Tanzania FSW-ART [25]	Community-based ART distribution can lead to higher ART initiation rates with continued ART use and better adherence after six months	The Tanzania Ministry of Health changed the national policy and issued a circular authorizing community-based ART initiation to key and vulnerable Populations
Senegal TnS [26]	HIV self-testing (Senegal TnS) is feasible	National AIDS program included HIV self-testing in the national HIV test and start strategy
Eswatini FAMCARE [27]	43.1% of children on ART were receiving a suboptimal Nevirapine-based regimen	The Eswatini Ministry of Health changed the policy and issued a facility memo to transition children and adolescents on Nevirapine-based regimens to better regimens (either Lopinavir/ritonavir-based or Efavirenz-based)
Eswatini PrEP modelling [28] Uganda PrEP modelling [29]	Modeling projected cost-effectiveness and impact of PrEP in various target populations	The Eswatini and Uganda Ministries of Health modified their choice of national priority target populations for PrEP in line with recommendations from the modelling

#### Project SOAR impact

The aim of Project SOAR studies was to strengthen programs that contribute to the global goals of ending the HIV epidemic through attainment of the 90–90-90 targets. The first 90 stands for ‘90% of all people living with HIV will know their HIV status’; the second stands for ‘90% of all people diagnosed with HIV infection will receive sustained antiretroviral therapy; and the third ‘90% of all people receiving antiretroviral therapy will have viral suppression’ [30]. Therefore, the long-term impact of Project SOAR studies would be changes in these proportions as a result of study findings: increases in the proportion of people who know their HIV status (first 90), increases in the proportion of people living with HIV who are on ART (second 90), and increases in the proportion of those on ART who are virally suppressed (third 90).

By definition, impact can only be observed after years or even decades of program work. There is a time lag between publication of research results and the utilization of these findings for policy or program change, and the corresponding reflection of these changes on population-based indicators. This makes it difficult to document the long-term impact of research utilization in the course of a six-year project [15, 31]. Therefore, a key limitation of this paper is that we are unable to present data on impact. In addition, population-level indicators of prevalence of knowledge of HIV status, ARV treatment, and viral-load suppression are impacted by other ongoing activities and programs. It is therefore challenging to attribute the influence of isolated research-utilization activities on overall indicator improvement. PEPFAR conducts population-based HIV impact assessments, to gauge and guide the entire HIV response in priority countries [32]. Therefore, assessing the long-term impact of Project SOAR research-utilization activities is beyond the scope of the project.

#### Quality and cost considerations

We enumerate the number of incidents of use of research findings; however, we do not discuss qualitative parameters, such as contextual factors influencing successful adoption of research, such as targeting the right stakeholders at the right time in the program cycle, and prevailing favorable political circumstances. While such a detailed discussion of each incident of use would be ideal, it is beyond the scope of this paper. However, further information about each reported incident of use is published on Project SOAR’s website and in peer reviewed journals, and we provide relevant citation throughout the text. We invite research organizations interested in replicating Project SOAR’s research-utilization interventions to review the Project SOAR publications we cite and learn about the program-specific contexts, bearing in mind that while these contexts may not be replicable, the concepts maybe transferable.

We also do not provide a costing of the research-utilization interventions that may have led to the research-use incidents that we report. Such information is not readily available because, as explained above, research-utilization activities were budgeted as integral components of each Project SOAR study, and it would require a detailed costing study to extract the research utilization costs. Such a study is beyond the scope of this paper. Nonetheless, the paper presents three major categories of illustrative budget items that could inform replication. One category is personnel, and here we mention a 100% Research-Utilization Advisor, a 100% Science Writer and a 50% Knowledge Management Specialist. The second category is inputs, listed in Table 2, including capacity strengthening workshops, a small grants program, international trips for the Research-Utilization Advisor, and research advisory committee meetings. A third budget category comprises briefs, presentations and meetings used to disseminate study outcome information, listed in Table 3. We believe these budgetary items can assist research programs to incorporate research-utilization activities into their research budgets.

## Discussion and conclusion

Project SOAR is dedicated to implementation science. As such, the project’s science (research studies) was undertaken specifically to inform the implementation of programs and policies. Therefore, research utilization was embedded in all phases of Project SOAR studies, from design through implementation and dissemination of findings. This essay presented the process Project SOAR used for research utilization and how it was documented and measured. Our discussion followed the project’s research-utilization logic model, which includes inputs, activities, outputs, outcomes (short-term and intermediate) and impact – not of the research studies, but of the utilization of findings from these studies.

To capture activities, outputs, and outcomes, the project developed prospective tools, such as the stakeholder engagement tool (Table 1). Using prospective tools, completed as the research-utilization activities progressed, enabled the researchers to capture ‘indicators as they emerge’ [15]. Prospectively collected indicators are more powerful than retrospective indicators, collected after a study is completed, because retrospective indicators may be prone to recall bias [33]. Collecting indicator data prospectively also allowed researchers to interact with stakeholders earlier and have more opportunities for utilizing research findings for program and policy improvements.

### Challenges to utilization of study findings

Research utilization, like any program activity, has cases of failure, or less than optimal success. In the case of research utilization this translates to instances where research findings are not used for decision making. Documentation of research use should also include non-use of findings and reasons why data were not used. Reasons for non-use vary and may include, for example, insufficiently sound research methodology, unconvincing study findings, infidelity of a tested intervention, failure of a tested pilot intervention, or instances where stakeholders are not optimally engaged in the research process or the interpretation of results [34].

From the stakeholder perspective, reasons for non-use of research findings can be that the findings do not align with a decision-making moment, that there is no mechanism within which to enact change, or that there is no budget to incorporate the change. This is one of the reasons to document research utilization in all phases of research and dissemination, to enable researchers and stakeholders to link research impact or non-impact to how data utilization was promoted [15].

Project SOAR required all study researchers to report on activities and stakeholder engagement every 6 months through the life of the project, thus providing information needed to further analyze what was more, or less, effective in generating research-findings use. Following is a discussion of some challenges that Project SOAR, and other research projects, could face in monitoring of research-utilization activities and outcomes.

#### Reporting bias

Documentation and monitoring of research utilization is susceptible to reporting bias. Most methods of data collection to monitor research utilization involve interviews with, or by, research generators and research users [14, 28, 35], and are therefore subject to recall bias as the respondents tend to recall only what worked. They may also be susceptible to social desirability bias, because respondents would want to please the interviewers, or they have a vested interest in showing the value of the investment made in research. In addition, the selection of instances of research utilization that are reported tends to be biased towards “high-impact rather than low-impact” research, based on the level of impact on programs or policies [14]. Routine reporting every 6 month, and supervision of the Research-Utilization Advisors in all project studies, helped reduce these biases in Project SOAR studies.

#### Attribution

Attributing a policy or program change to a specific research finding is difficult and often impossible. Research-utilization activities are usually not implemented as randomized controlled interventions; results of a study are simply presented to decision makers without an experimental design to exclude other factors that could influence decision-making. Such factors include political forces and other external policy influences, good fortune, other studies, and the magnitude of the problem being addressed by the research findings [14, 15, 26]. Further, there is usually no counterfactual; meaning that there is no way to tell what would have happened had the study not been conducted [29], or had the results not been presented to these particular stakeholders, at that specific time, in exactly that manner and setting [28].

The closest we can get to attribution is when the change in policy or practice is made after the dissemination of findings to the specific stakeholders who made the decision. For example, after Project SOAR investigators presented study results to the national AIDS program in Senegal that showed the feasibility of HIV self-testing [26], the national AIDS program included self-testing in the national HIV test-and-start strategy. We can posit that without the Project SOAR study, the national AIDS program may have still included self-testing in its strategy; on the other hand, it could also be argued that Project SOAR’s feasibility study provided confidence to the national AIDS program to implement self-testing.

Searles et al. (2016) recommend that as part of the study protocol-development process, researchers should assess the policy landscape related to the guidelines or policies that the study is intending to influence. Such information can ensure that the correct stakeholders are identified, and give support to the case for attribution when corroborated with the fact that the policy or program change was made after the research findings were disseminated to the specific stakeholders who made the change [4].

#### Unpredictable time lag

The time between completion of research activities and utilization of its findings varies widely. Some study results are used immediately after a study is completed, even before the results are published; or it may take months or even years for results to affect policy or program change. For example, in Project SOAR’s study on the feasibility of HIV test-and-start in Namibia [20], the implementing partner used the data before the results were disseminated at a national meeting. The implementing partner then reported that preliminary study data were very compelling, so they went ahead and implemented some of the recommendations (such as differentiated ART for youth and reduction in frequency of clinician consultations for stable patients).

However, quite often research findings are not used for years or even decades after they are published [28]. With the possibility of infinite time lag between publication and utilization of research findings [15] it becomes difficult to document when study results have been used because often, after the study ends, the interested parties do not have the resources to continue monitoring and documenting instances of utilization. It is, therefore, impossible to conclude that a study’s results have not, or will never be, utilized. For this reason, Project SOAR investigators developed data use plans (in collaboration with stakeholders) which include the identification and cultivation of champions who will continue promoting the use of study findings at opportune moments, even after the end of the project. For example, in Malawi, Project SOAR study of the DREAMS program found a high prevalence of genital herpes among adolescent girls and young women, which could influence the go/no-go decision about providing PrEP to beneficiaries. Our Champion planned to follow up by attending the prevention technical working group and discussing our results, together with other data used for making the go/no-go decision.

## Recommendations

Funders of implementation research are increasingly seeking to understand the impact of research to assess returns on their investments [4]. Moreover, as funding to support some areas of research wanes or remains stagnant, the need to document the value of investing in research by its’ effect on improved programs and policies becomes increasingly important.

While monitoring of research utilization is not a widely established component of research, Project SOAR’s experience has shown that researchers can use a simple set of tools and guidance to record activities they conduct, to systematically ensure that stakeholders are aware of research findings and the intent and commitment from stakeholders to take programmatic or policy decisions. Based on Project SOAR’s experience, we propose the following recommendations:

1. Research organizations should require researchers to routinely use research-utilization guidance and reporting tools for all significant research investments;
2. At the design phase of each study, assess the relevant policy landscape, to ensure that the appropriate stakeholders are identified and engaged, and to build the case for attribution;
3. Collect and use stakeholder engagement and research-utilization data progressively, throughout all phases of the study, starting at the design phase. This can help improve stakeholder buy-in to research findings, development of appropriate recommendations and minimize bias; and
4. Implement systematic research-utilization activities, including identifying and coaching in-country champions to promote study findings over time and to develop data use plans that define a roadmap for research-utilization activities to guide the champions to promote study findings at future opportunities after study results are disseminated.

## Data Availability

Not applicable.

## Abbreviations

ART: Anti-retroviral therapy
CDC: Centers for Disease Control and Prevention
DREAMS: Determined, Resilient, Empowered, AIDS-free, Mentored, and Safe
PEPFAR: President’s Emergency Plan For AIDS Relief
PrEP: Pre-exposure prophylaxis
SOAR: Supporting Operational AIDS Research
STI: Sexually transmitted infections
UNAIDS: Joint United Nations Programme for HIV/AIDS
USAID: United States Agency for International Development

## References

[1] Estabrooks CA, Wallin L (2004). Where do we stand on the measurement of research utilization? Paper prepared for the 4^th^ Annual Knowledge Utilization Colloquia (KU04), Belfast, Northern Ireland.

[2] United States Agency for International Development (USAID). Improving HIV and AIDS programming through implementation science. Washington, DC: USAID; 2014. https://www.usaid.gov/global-health/health-areas/hiv-and-aids/technical-areas/implementation-science

[3] Mallonee S, Fowler C, Istre GF (2006). Bridging the gap between research and practice: a continuing challenge. Brit Med J..

[4] Searles A, Chris DC, Attia J (2016). An approach to measuring and encouraging research translation and research impact. Health Res Policy Syst..

[5] Centers for Disease Control (CDC). Practical use of program evaluation among sexually transmitted disease (STD) programs. Atlanta: Division of STD Prevention, National Center for HIV/AIDS, Viral Hepatitis, STD, and TB Prevention, Centers for Disease Control and Prevention; 2014. https://www.cdc.gov/std/program/pupestd.htm

[6] Project SOAR (2016). RU Guide. Project SOAR’s approach to research utilization, Project SOAR 2016.

[7] Project SOAR (2018). Lesotho IMPROVE. Evaluating a multidisciplinary integrated management team intervention to improve maternal and child outcomes and HIV service uptake and retention in Lesotho. Project SOAR activity brief.

[8] Kalibala S, Nutley T. Engaging stakeholders, from inception and throughout the study, is good research practice to promote use of findings. AIDS Behav. 2019. 10.1007/s10461-019-02574-w Published online 3 July 2019. 10.1007/s10461-019-02574-w PMC6773669 31270641

[9] Kalibala S (2018). Improving HIV programs: Developing in-country capacity to generate and use Data.

[10] Project SOAR (2019). Q&A with Sam Kalibala, Director of Research Utilization, answers questions about SOAR’s strategy to promote research uptake.

[11] Kalbarczyk A, Davis W, Kalibala S, et al. Research capacity strengthening in Sub‑Saharan Africa: Recognizing the importance of local partnerships in designing and disseminating HIV implementation science to reach the 90–90–90 goals. AIDS Behav. 2019. Published online 16 May 2019. 10.1007/s10461-019-02538-0. 10.1007/s10461-019-02538-0 PMC6773819 31098746

[12] Sumner A, Crichton J, Theobald S, Zulu E, Parkhurst J (2011). What shapes research impact on policy? Understanding research uptake in sexual and reproductive health policy processes in resource poor contexts. Health Res Policy Sy..

[13] Raftery J, Hanney S, Greenhalgh T, Glover M, Blatch-Jones A (2016). Models and applications for measuring the impact of health research: Update of a systematic review for the Health Technology Assessment Programme. Health Technol..

[14] Makkar SR, Brennan S, Turner T, Williamson A, Redman S, Green S (2016). The development of SAGE: A tool to evaluate how policymakers engage with and use research in health policymaking. Res Evaluat..

[15] Penfield T, Baker MJ, Scoble R, Wykes MC (2014). Assessment, evaluations, and definitions of research impact: A review. Res Evaluat..

[16] Project SOAR (2018). Kenya and Uganda pediatric case finding. Active pediatric HIV case finding in Kenya and Uganda.

[17] Project SOAR (2019). Zambia Project YES. Youth living with HIV in Zambia: interpersonal violence, self-stigma, and viral suppression. Project SOAR Results Brief.

[18] Rodríguez DC, Paina L, Wilhelm J (2019). Evaluating the impact of PEPFAR’s geographic prioritization on centrally supported health facilities. Project SOAR final report.

[19] Settergren SK, Mujaya S, Rida W (2018). Cluster randomized trial of comprehensive gender-based violence programming delivered through the HIV/AIDS program platform in Mbeya Region, Tanzania: Tathmini GBV study. PLoS ONE.

[20] Project SOAR (2019). Namibia TnS. Viral load testing: Room for improvement in Namibia’s antiretroviral treatment services. Project SOAR Results Brief.

[21] Project SOAR (2018). Tanzania FSW/FP. Assessing family planning and safer conception needs and services among female sex workers living with HIV in Dar es Salaam. Project SOAR Final Report.

[22] Lisa B, Musoke P, Etima M (2017). Increasing pediatric HIV disclosure to children in Uganda. Project SOAR Results Brief.

[23] Project SOAR (2018). Global Fund TA. Strengthening capacity to use data to inform HIV responses for key populations. Project SOAR Activity Brief.

[24] Mensch BS, Erica SH (2017). Rates of HIV and HSV-2 among young people in Machinga, Malawi. Project SOAR Results Brief.

[25] Project SOAR (2019). Tanzania FSW-ART. Community-based delivery of antiretroviral treatment for female sex workers in Tanzania: high levels of initiation, use, and adherence. Project SOAR Results Brief.

[26] Project SOAR (2019). Senegal TnS. Can HIV Self-testing Help Reach Those at Risk for HIV and Not Accessing Traditional Testing Services in Senegal? Project SOAR Results Brief.

[27] Chouraya C, Ashburn K, Khumalo P (2019). Association of antiretroviral drug regimen with viral suppression in HIV-positive children on antiretroviral therapy in Eswatini. Pediatr Infect Dis J..

[28] Project SOAR (2019). Eswatini PrEP modelling. Oral Pre-Exposure Prophylaxis Modeling Results: Eswatini, Results to inform Ministry of Health’s PrEP scale up. Project SOAR Results Brief.

[29] Project SOAR (2019). Uganda PrEP modelling. Oral Pre-Exposure Prophylaxis Modeling Results: Uganda, Results to inform Ministry of Health’s PrEP scale up. Project SOAR Results Brief.

[30] UNAIDS (2020). 90-90-90: An ambitious treatment target to help end the AIDS epidemic.

[31] Frank C, Nason E (2009). Health research: measuring the social, health and economic benefits. Can Med Assoc J..

[32] Centers for Disease Control (CDC) (2019). New PHIA survey data show critical progress towards global HIV targets.

[33] Straus SE, Tetroe J, Graham ID, Zwarenstein M, Bhattacharyya O, Shepperd S (2010). Monitoring use of knowledge and evaluating outcomes. CMAJ..

[34] Banzi R, Moja L, Pistotti V, Facchini A, Liberati A (2011). Conceptual frameworks and empirical approaches used to assess the impact of health research: an overview of reviews. Health Res Policy Sy..

[35] Proctor E, Silmere H, Raghavan R (2011). Outcomes for implementation research: Conceptual distinctions, measurement challenges, and research agenda. Adm Policy Ment Health..

